# Old(er) care home residents and sexual/intimate citizenship

**DOI:** 10.1017/S0144686X15001105

**Published:** 2015-10-14

**Authors:** PAUL SIMPSON, MARIA HORNE, LAURA J. E. BROWN, CHRISTINE BROWN WILSON, TOMMY DICKINSON, KATE TORKINGTON

**Affiliations:** * Department of Applied Health and Social Care, Edge Hill University, Ormskirk, Lancashire, UK.; † Faculty of Health Studies, University of Bradford, Bradford, UK.; ‡ Department of Psychological Sciences, University of Manchester, Manchester, UK.; § Department of Nursing and Midwifery, University of Queensland, St Lucia, Brisbane, Australia.; || Department of Nursing, Midwifery and Social Work, University of Manchester, Manchester, UK.; ¶ Valuing Older People, Manchester City Council, UK.

**Keywords:** ageism, care homes for older people, exclusion, heteronormativity, intimacy, sexuality

## Abstract

Sexuality and intimacy in care homes for older people are overshadowed by concern with prolonging physical and/or psychological autonomy. When sexuality and intimacy have been addressed in scholarship, this can reflect a sexological focus concerned with how to continue sexual activity with reduced capacity. We review the (Anglophone) academic and practitioner literatures bearing on sexuality and intimacy in relation to older care home residents (though much of this applies to older people generally). We highlight how ageism (or ageist erotophobia), which defines older people as post-sexual, restricts opportunities for the expression of sexuality and intimacy. In doing so, we draw attention to more critical writing that recognises constraints on sexuality and intimacy and indicates solutions to some of the problems identified. We also highlight problems faced by lesbian, gay, bisexual and trans (LGB&T) residents who are doubly excluded from sexual/intimate citizenship because of ageism combined with the heterosexual assumption. Older LGB&T residents/individuals can feel obliged to deny or disguise their identity. We conclude by outlining an agenda for research based on more sociologically informed practitioner-led work.

## Introduction

It seems that sex is only for the young. This is evident in the lack of media images of older people as sexual beings (Garrett 2014) and ‘gift’ cards that ridicule their assumed lack of sexual, physical and cognitive capacity (Bytheway 1995). Stereotypes govern thinking of ageing sexuality as either ‘inhibited or inactive’ (Mahieu, Anckaert and Gastmans 2014: 1) and do not just homogenise but also situate older people outside the youthful sexual norm. Yet, people do not necessarily cease desiring when pronounced old (Gott 2005) or when they need to live in a care home. Indeed, Bauer *et al.* (2012) have identified that residents can express a range of responses towards sex and sexuality from denial to nostalgia and continuity. Whilst health problems can encourage redefinition of sex (Mahieu, Anckaert and Gastmans 2014), intimacy remains important until the end of life (Kuhn 2002). Further, there remains a widespread prudishness concerning the sexuality of older people; a subject commonly ignored (Garrett 2014; Gott 2005; Hafford-Letchfield 2008; Villar *et al.* 2014) or else framed as a problem to be managed (Doll 2012). This indicates the workings of ageism fraught with stigma, feelings of disgust at the thought or sight of frail bodies, and is implicated in the infantilisation of older people considered asexual or in need of protection from their desires by carers and relatives (Gott 2005; Hockey and James 1993).

This narrative review of scholarship has emerged in response to the persistence of ageist attitudes about sexuality and intimacy (Bauer *et al.* 2012; Doll 2012; Gott 2005; Villar *et al.* 2014; Wornell 2014). Such attitudes persist despite nearly half a century of thinking about holistic needs assessment (Katz and Stroud 1963). Along with emphasis on the individual needs and wishes of service recipients, holistic assessment and care are enshrined in the National Health Service and Community Care Act 1990. Thinking has developed more than 25 years since the arrival of community care legislation in the direction of practices that promote personalisation of services and individual control of delivery of care, including budgets (Carr 2013; Department of Health 2007*a*). Yet, older people's needs relating to sexuality and intimacy appear designed out of care systems and are largely absent from ageing and care policy (Garrett 2014; Hafford-Letchfield 2008). Tellingly, the proportion of single rooms in privately owned care homes accommodating older people increased from 60 per cent in 1989 to 94 per cent in 2013 (Laing 2014). This situation could in some parts of the United Kingdom (UK) reflect less an obsession with profit margins than national minimum standards that urge that by 2010, 85 per cent of rooms be devoted to single occupancy (Care and Social Services Inspectorate, Wales 2004). It is also worth bearing in mind that it is rare for couples to be admitted to a care home at the same time and some couples, given health status, may prefer or need to sleep alone. In such cases, homes could try to place couples in adjoining rooms. However, the structure of environments combined with the above-identified silence among academics, practitioners and policy makers generally reinforce older people's exclusion from what Plummer (1995) calls ‘sexual/intimate citizenship’ – a concept that is elaborated below but for now refers to a valid identity as a sexual/intimate being.

We focus on care home residents because, compared with those living more independently, their opportunities to express themselves as sexual and/or intimate beings are much more likely to be restricted (Bauer *et al.* 2014; Doll 2012; Phillips and Marks 2008; Villar *et al.* 2014; Wornell 2014). Privacy can be more often compromised here (Bauer *et al.* 2012) – sometimes necessarily so in cases of urgency or emergencies. The idea of ‘privacy’ is more problematic and takes on a different hue in the context of adult care homes. Although residents' rooms are understood as private/personal space, staff may feel they have a legitimate right to access this space for care delivery, resulting in difficulties for residents in maintaining choice and autonomy (Eyers *et al.* 2012). Equally, there are communal areas where privacy around sexuality and other matters might be further compromised. It is worth noting that care staff and residents have different orientations to the spaces of care. For the former, they are workplaces that require professional negotiation of empathy and avoiding over-involvement (Green, Gregory and Mason 2006). In contrast, for residents, entry to a care home requires adjustment to changes in their abilities, social support structures, relationships and their connections with significant others and community (Cook 2006; Eyers *et al.* 2012; Hutchinson *et al.* 2011). Residents are obliged to renegotiate meanings, identities and relationships in these new contexts (Cook, Thompson and Reed 2014): in other words, the whole basis of their ontological security – the ability to be oneself with familiar others (Wiles *et al.* 2012). This is particularly important for lesbian, gay, bisexual and trans (LGB&T) individuals. Note that ‘trans’ encompasses a range of identities. Trans identity can be claimed variously by people who cross-dress, are receiving hormonal treatment (but may still wish to retain a penis or vagina/uterus) or have undergone full re-assignment surgery to embody their preferred gender. Some individuals might identify with the gender with which they feel most comfortable rather than that which they might be thought (sometimes mistakenly) to represent. Yet other individuals might identify as a non-binary form of gender that is neither male nor female (*see* Simpson 2015 for a fuller explanation).

Our argument in this article is threefold. First, we contend that concern in the generic literature on care homes with prolonging physical and/or psychological autonomy (*see* Kane *et al.* 2003; Mozley *et al.* 1999;) overshadows concerns with sexuality and addresses autonomy in limited ways that reinforce residents' and older people's exclusion from sexual citizenship (Bauer 1999; Bauer *et al.* 2012). Second, a fair degree of extant literature on ageing and care homes treats sexuality in limited sexological ways, *i.e.* that emphasise continuing (hetero)sexual functioning over emotional content (Trudel, Turgeon and Piché 2000) and thus reduce sexuality to a book-keeping approach that concerns who is still having sex in changed circumstances and how often. We note, however, how some more sociologically informed work influenced by feminist–humanist thinking has highlighted the problem of ageism and gerontophobia – ageing/old age as something to be feared – or ageist erotophobia – disgust at the thought of ageing body-selves as sexual (*see* Hafford-Letchfield 2008; Hockey and James 1993). Whilst acknowledging older people's exclusion from the sexual imaginary, these contributions highlight the discursive and structural constraints on expression of sexuality whilst offering some solutions at the level of policy and practice (Hafford-Letchfield 2008; Villar *et al.* 2014). Third, we draw attention to the problem of heteronormativity and homo-/lesbo-/bi-/transphobia (Stein and Almack 2012). The former takes heterosexuality as the benchmark of sexual citizenship and the latter refers to fear-based ignorance that can induce hostility and result in exclusion of older LGB&T individuals. Residents can feel obliged to go back into ‘the closet’ and become ‘twice hidden’ because of the influences of ageism interacting with the heterosexual assumption and homo-/lesbo-/bi-/transphobia (Rainbow Project and Age Northern Ireland 2011; Willis *et al.* 2013).

To contextualise the discussion, we briefly explain the approach and methods behind our literature search, discuss statistics on later life and care homes, and offer definitions of key terms – sexuality, intimacy and ‘sexual/intimate citizenship’. We do not theorise ageing or later life, as these have been well documented elsewhere (*see* Bengtson 1999; Johnson, Bengtson and Coleman 2005) and are outside the scope of this paper. Substantively, the main section then reviews the literature on care homes for older people, which takes in the work addressing older people generally and the double exclusion of LGB&T residents. The concluding section summarises key themes and outlines an agenda for research. This includes reference to: more sociologically informed work (*e.g.* Hafford-Letchfield 2008) that identifies practical solutions to meeting older care home residents' needs concerning sexuality and intimacy; and a brief discussion of dimensions of care for and attitudes towards older people in less economically developed societies.

## Literature search

This is a narrative review that aims to ‘summarise, explain and interpret evidence on a particular topic/question’ using qualitative and/or quantitative evidence (Mays, Pope and Popay *et al.* 2005: 11). As there is limited evidence on this subject, a systematic strategy was undertaken to ensure that as many papers as possible were located. Specifically, the process involved searching: (a) Google Scholar – which yielded journal articles, monographs, book chapters, government and third-sector reports whose reference sections were also searched for further sources; (b) texts on ageing and/or care known to the research team, with reference lists examined for relevant references; (c) a search through key journals, national and international, covering ageing/social gerontology, nursing (age-related and generic), sociology, psychology and social policy. Further readings were recommended when we consulted on a developed draft of this article with fellow academics who have undertaken research on ageing sexuality.

## Context

Generally, people are living longer and will face higher risks of failing health, especially in the last few years of life (Dunnell and Office for National Statistics (ONS) 2008) when they are more likely to need care home accommodation. But, longevity reflects dominant forms of social inequality and differences. In order to appreciate who we are talking about in this article, and given that older people and residents are different in various ways, it is important to consider who is most likely to survive into later life and thus be more likely to need accommodation in a care home. Longevity largely reflects forms of social inequality along lines of gender, ethnicity and social class.

Table 1 shows that old age is affected by ethnicity and is ‘feminised’ (Arber and Ginn 1991). Government statistics show that the ratio of men to women aged 65 or over in the UK in 2010 was 100:154. Men aged 65 or over amount to nearly two-thirds of the number of women surviving to or beyond this age (ONS 2011*a*). This discrepancy is partly attributable to how men are socialised to take more risks – tending to work in more dangerous occupations – and to be less vigilant than women about their health (Peate 2004). Among those aged 85 and over, who are more likely to be accommodated in care, women outnumber men by a factor of 2:1 (ONS 2011*b*). Longevity and class correlate highly: individuals from wealthier sections of society are more likely to live longer. Those in the poorest wealth quintile have an overall 56 per cent greater chance of mortality across all age groups than individuals in the wealthiest quintile (Nazroo, Zaninotto and Gjonca 2008).

**Table 1.** Gender, race and average lifespan

Gender and race	Average lifespan (years)
White British men	76.4
White British women	80.3
Afro-Caribbean British men	75.3
Afro-Caribbean British women	81.4

*Source*: Based on data from Wohland *et al.* (2014).

In terms of statistics on care homes for older people, Census data show that of the 10.3 million people aged 65 or over in the UK, 4.5 per cent were accommodated in a communal home (ONS 2011*b*). It is worth noting that this figure represents more than 500,000 people. It has also been estimated by campaigning group Stonewall (Stonewall and Taylor 2012), that older LGB&T people aged 55 and over represent a sizeable minority equivalent to the population of Birmingham, (just over 1,101,000 people), yet Knocker (2012) reminds us that their views are seldom sought. Also, the majority of care homes for or accommodating older people are largely privatised: of the nearly 500,000 beds available in the UK, 350,000 (70 per cent) are situated within independent, for-profit residential homes (Laing 2014). Individuals aged 85 or over represent 58 per cent of the population in care homes for older people (ONS 2014). About one in ten men and one in five women aged 85 and over live in a communal establishment (ONS 2011*b*) and women outnumber men here by a ratio of 2.8:1 (ONS 2014). Care homes also accommodate some ‘younger old’ people who need care by virtue of conditions like Parkinson's disease or early onset of a dementia. It is estimated that about two-thirds of care home residents experience some degree of dementia (ONS 2011*b*). In summary, care homes are more often populated by women and middle-class people surviving into the ninth decade.

## Definitions: sexuality, intimacy and ‘sexual/intimate citizenship’

We define ‘sexuality’ as a social process that includes the quality of being sexual and sexual identification whether gay, straight, bisexual, queer or ambiguous (*see* also Jackson and Scott 1996). Sexual identity also concerns how we express ourselves in terms of emotions, desires, beliefs, self-presentation, and the kind of activities and relationships we engage in and the ways in which we engage in them. Following Doll (2012), we view sexuality as multi-dimensional – as constituted by biological, *e.g.* bodily sensations that we interpret as sexual; psychological, *e.g.* emotions and cognition; and cultural/social influences. The latter encompass how we feel/think about our bodies – as manifest in going to the hairdressers, dressing up smart or flirting – as well as needs for touch and emotional connection. It is also influenced by norms governing who can talk about sex and be sexual and/or intimate and in what contexts.

The expression of sexuality is heavily influenced by gender ideology interacting with generational factors. For instance, loss of sexual capacity in later life is thought to be more difficult for men to manage given that they fear the loss of equality or dominance within a relationship and are generally more reluctant than women to talk through sexual and/or relationship problems (O'Brien *et al.* 2012). In contrast, the sexuality of many old women will have been constrained by moral imperatives of being a good wife and mother, though women now in middle age, born during or since the post-war baby-boom, will have encountered the influences of feminism (Rowbotham 1999). Social critique by the women's and lesbian and gay movements from the 1960s onwards paved the way for greater autonomy over fertility and expression of sexuality (Rowbotham 1999). Sexuality also enmeshes with influences of age/generation. Because sexuality for women is associated with youthful beauty, this tends to exclude older women (Doll 2012) and the double-standard persists whereby older females especially face moral censure for acting age-inappropriately, *e.g.* for being morally ‘loose’ (Rosenthal 1990; Wilcox 1997). Whilst older males expressing desire – appropriately or otherwise – can be stereotyped as a ‘chip-off-the-old-block’ or sometimes ‘dirty old men’, older women who are sexually assertive are commonly seen as breaching a legitimate ageing femininity that demands decorum and passivity (Brown 1992).

When we speak of ‘intimacy’, we refer to involvement in close personal relationships. Like sexuality, intimacy is multi-dimensional – affected by the mutually influencing differences of age/generation, gender, class and ethnicity. It is also gendered: if men tend to define it more in physical terms, women emphasise more its emotional content (O'Brien *et al.* 2012). Further, Ehrenfeld *et al.* (1997) have argued that intimacy covers a spectrum of emotions, needs and activities ranging from feelings of caring, closeness and affection that go with companionship and that may or not involve sexual feelings or activity through to ‘romance’ where we mark out or ‘idealise’ a person(s) in our affections. In this formulation, at the other end of the spectrum lies ‘eroticism’ that involves strong sexual excitement and desire and, according to Ehrenfeld *et al.* (1997), when reciprocal, is more likely to encompass sexual activity. Closeness, romantic feelings and sexual activity can involve different degrees and kinds of touch, though the lines between each category above might be blurred or combined (or not) in relationships. It is possible to distinguish intimacy from sexuality – the former is seen as much broader than sexual activity and can invoke a deeper, mutual understanding developed *over time*, involving gentler forms of tactility. Indeed, it has been suggested that older people are redefining sexuality as intimacy (Doll 2012). This could reflect pragmatism in the face of loss of capacity or agency in the form of resignification of the meanings of sex and sexuality. Whilst the notion of a continuum entertains gradations of feeling and experience, we question the implied mutual exclusivity in the polarisation of affection and eroticism. Although the distinction between sex and intimacy, or rather tenderness, is meaningful to those who make it, if we believe in a plurality of experience, then any adequate theorisation of intimacy should recognise the nuances in-between tenderness and sexual activity and the points of distinction *and* overlap between these experiences.

Moreover, Evans (1993) describes sexual citizenship as intrinsically relational and constituted by intersecting moral (cultural/discursive) and socio-economic (structural or class) dimensions. Whilst this baseline definition is useful, Plummer's (1995) definition is particularly germane to our argument. In this formulation, ‘sexual/intimate citizenship’ is commonly articulated in and through claims to validity by minoritised groups – those seen as ‘sexually different’ and seeking ‘control … over one's body, feelings, relationships: access to representations, relationships, public and socially grounded choices … about gender identities’ (Plummer 1995: 151). This statement could be extended beyond LGB&T individuals to involve older care home residents and older people generally who constitute a seldom-heard population, and especially on sexual matters. Plummer's thinking is useful in that it conceives of sexual/intimate citizenship as part of a range of possibilities that are worthy of recognition and respect. Indeed, this thinking is reflected in legislative change which has legalised same-sex marriage and undergirds the Single Equalities Act 2010, which offers legal protection to LGB&T individuals and groups as well as to older people in terms of their access to goods, services and the labour market/employment.

## Sexuality and intimacy in care homes

### Exclusion: ageism and the organisation of care

Scholarship on care homes appears dominated by bio-medical paradigms (Bauer 1999). Concerns with prolonging physical independence by means of assistive devices or technologies (Miskelly 2001) or the avoidance of falls or hip fractures (Oliver *et al.* 2007) have tended to overshadow residents' or older service users' needs relating to sex, sexuality and intimacy. Such needs are commonly assumed to be outside the primary care-giving role because they are not thought vital to the maintenance of bodily functions (Bauer 1999) and are considered too private or personal. This basic approach to care – premised on a bed-and-body principle – goes against the notion of needs as holistic. By this reckoning, old(er) people and residents are deemed worthy of a decent quality of life but one that precludes sexual relations and/or intimacy. Whilst vital to a sense of wellbeing, biological and psychological paradigms and the practices they support are insufficient in themselves to address the holistic requirements of old(er) people, whether living in residential care or with support in their own homes. In line with thinking implicit in Bauer *et al.* (2012) and Hafford-Letchfield (2008), we contend that the self-empowerment of care home residents and older individuals will depend equally, if not more, on critical thought and practice concerning sexual/intimate citizenship by those working for and with older people.

What work does exist on sex, sexuality, intimacy and older residents and older people generally appears to reflect a sexological bias that involves focus on sexual functioning – how to continue with sexual activity in the context of physical loss. As Gott (2005) points out, attention to the sexuality of older people appears fixated on quantifying sexual activity; in particular, which older heterosexual people are having sex and how often. Some of this work, which is largely North American and commonly derived from survey and/or clinically based investigations, not only medicalises ageing sexuality (Hodson and Skeen 1994) but also reinforces ageism, sexism and heteronormativity. Specifically, an article by Trudel, Turgeon and Piché (2000) addresses the sexuality of older people predominantly in physiological, functional and rather emotion-free terms. Indeed, their analysis focuses largely on capability for heterosexual coition, which exemplifies a book-keeping approach to ageing sexuality. Despite the fact that the authors present data on sex in later life that show it to be fairly commonplace and important in maintaining self-esteem, their view of ageing sexuality as synonymous with physical decline and hampered by morbidity homogenises older people and the ageing experience. Such thinking ignores the inventiveness of older people as diverse, experienced, adaptive sexual agents (Mahieu, Anckaert and Gastmans 2014). It also overlooks the possibility that some individuals will have developed cognitive, emotional and political resources through experience of ageing or ‘ageing capital’ (Simpson 2014) to challenge existing gender and sexual norms that decree that they should be discreet about or even avoid expressing their sexuality.

It appears that ageist erotophobia – which refers to deep-seated anxieties about older people as sexual beings or failure to see them as such – is implicated in lack of awareness of the diverse lived experiences and needs of older people. This is commonly expressed in discomfort or even disgust at the idea of older people as sexual beings and sexual activity among this social group is commonly considered, ‘rare, astonishing and ridiculous’ (Hodson and Skeen 1994: 223). Anxieties about the sexuality of older people can work to erase their sexual histories. Indeed, sex, sexuality and intimacy are commonly seen as irrelevant to ageing identities, individuals' sense of belonging and citizenship (Bauer 1999; Bauer *et al.* 2014; Doll 2012; Gott 2005; Hafford-Letchfield 2008; Hockey and James 1993; Villar *et al.* 2014). It has also been suggested that such attitudes are influenced as much by an underlying fear of mortality (Hodson and Skeen 1994). This appears to be a curiously neglected topic in itself and could reflect that old age is taken as synonymous with death – social and actual (Gilleard and Higgs 2000).

Anxieties about the sexuality of residents and older service users persist despite the existence of useful guidance documents by the Department of Health (2007*b*) and the Commission for Social Care Inspection (2008), both of which aim to foster good practice in assessment, person-centred planning and self-directed services in relation to LGB&T individuals. Furthermore, Local Government Association (2014) guidance on safeguarding cautions against desexualisation and infantilisation of individuals receiving care. The Royal College of Nursing (2011) focuses on ways of addressing sex and sexuality in residents'/older people's care plans and care homes' policies. The latter document is a practical resource that contains case studies to guide professionals in supporting residents with varying sexual needs. The report identifies lack of staff training, embarrassment around sexual issues, organisational cultures within homes that disregard sexuality and religious influences as significant inhibitors to addressing sexuality (Royal College of Nursing 2011). The Independent Longevity Centre, UK (2011) has also provided guidance on intimacy and sexuality in relation to individuals affected by dementia, which aims to support action at both institutional/policy and practice/interpersonal levels. In an Australian context, Bauer *et al.* (2014) have devised a toolkit to enable care staff to address residents' needs relating to sex, sexuality and intimacy which could be adapted for other countries.

There are, however, indications that various forms of guidance from Government and professional organisations are not being translated into the awareness and practices of professional carers. But why might this be? A qualitative meta-synthesis by Rushbrooke, Murray and Townsend (2014) based on 17 articles concerning care-givers' responses to sexuality and adults with intellectual disabilities describes care-givers' responses that contribute to such a situation, which could apply to people with dementia/compromised cognitive capacity. The authors identify the following factors as significant: (a) uncertainty around lack of competence and training in broaching sexual matters; (b) presumption of residents' asexuality clashing with pressures to view them as sexual beings; (c) difficulties in distinguishing sexually motivated expressions and enabling sexual activity; and (d) having to negotiate between safeguarding and enabling, which is thought to require constraints on the expression of sexuality. We would contend that age-inflected erotophobia is at the root of the kind of thought and practice identified by Rushbrooke, Murray and Townsend (2014).

Given the shift within the UK towards private care home provision, we need to consider how levels of funding could affect quality of service which would include staff awareness of needs relating to sexuality. It appears that profit margins have diminished considerably among all but one UK corporate provider of care homes for older people and that the private sector, which is: ‘not bound by public sector pay agreement … undercuts the public sector by paying staff close to the minimum wage’ (Laing 2014: 9). Lower rates of pay to fulfil the basic bed-and-body model of care, under-staffing and under-investment in staff development can mean that staff experience stress-inducing workloads and are given little training for roles that demand high degrees of social, professional and decision-making skill (Carr and Joseph Rowntree Foundation 2014). It has also been concluded that: ‘some care workers have strong personal skills but weaker literacy and numeracy skills, which can make the higher level training required for progression problematic’ (Carr and Joseph Rowntree Foundation 2014: 3).

The circumstances just described could affect care staff's self-esteem, compromise the quality of care (Lewis and West 2014) and result in high staff turnover (Cangiano *et al.* 2009). In turn, this leads to inconsistencies in care and lack of opportunity to build relationships with residents (Han *et al.* 2014) – something which is pivotal to addressing sexuality and intimacy. It appears that this situation is complicated by the arrival of migrant workers, largely female, in the care sector from African and Eastern European accession countries (McGregor 2007). Although migrants often arrive with good levels of education and are sometimes over-qualified, they frequently experience poor training opportunities (McGregor 2007). Some professional carers moving into a new culture may need help with more idiomatic communication in the home language and, more importantly, training to develop a fuller understanding of the complexities of British cultures and the UK regulatory context (Cangiano *et al.* 2009). Cultural differences in tolerance of sexual expression in various groups (Anderson 2007) might also create difficulties when moving to a country with different norms and values. Some individuals can arrive with deep-seated prejudices about how older people (women especially) should behave and with feelings of hostility towards LGB&T individuals (Knocker 2012), all of which will need addressing.

### LGB&T residents: ageism and heteronormativity/cisgenderism

Until more recently, institutionalised homophobia and cisgenderism meant that: ‘older LGB&T people were overlooked in health and social care legislation, policy, research, guidance and practice, which assume service users are heterosexual’ (Ward *et al.* 2010: 5). By cisgenderism, we refer to the prevailing norm that valid forms of gender identity and expression should align with the gender ascribed at birth or into which individuals have been socialised (Simpson 2015). Indeed, there is a paucity of work in the UK dedicated to the distinct care needs of older LGB&T residents (Willis *et al.* 2013). Such work is urgent when we consider that care staff can report being even more discomforted by expression of same-sex intimacy (Archibald 1998). Besides, a study by Heaphy, Yip and Thompson (2003) found that only 35 per cent of their study participants believed health professionals to be positive towards LGB&T service users and only 16 per cent of their respondents trusted health professionals to be knowledgeable about varied LGB&T lifestyles. Much of this has worrying consequences in light of LGB&T individuals' underuse of end-of-life care services (Stein and Almack 2012). The authors attribute this state of affairs to subtle forms of indirect discrimination, given lack of: awareness among staff; LGB&T-specific training; suitable images in service information; and failure to differentiate services. In the latter case, this can involve failure to differentiate between gay men themselves (not uniformly white), let alone between LGB&T individuals and an approach based on ‘treating them all the same’, which entails presupposition of heterosexuality and imposition rather than choice in service provision (National Council for Palliative Care and Consortium of Lesbian, Gay, Bisexual and Transgendered Voluntary and Community Organizations 2012). Nonetheless, homogenising attitudes and practices persist despite guidance in a UK context from Ward *et al.* (2010) on meeting the health and care needs of older LGB individuals and the Government-driven National Service Framework for Older People that urges culturally appropriate services that reflect ‘the diversity of … populations' (Department of Health 2001: 4).

It is not surprising then that older LGB&T residents experience more complex forms of prejudice and discrimination on the grounds of age enmeshed with gender/sexual difference (*see* Willis *et al.* 2013). Also, Westwood (2012) has highlighted the need to look at the intersections of ageing sexuality, including heterosexuality, with class and race and, we would add, differences of biography. Research by Willis *et al.* (2013), based on surveys, focus groups with professionals and older LGB people (trans was thought to require separate treatment), and in-depth interviews with LGB people aged 50–70, indicates that professionals commonly fail to recognise LGB individuals or do not have the awareness to gather this information sensitively. Not only did study participants state that they would have to surrender their sexual citizenship on entering a care home but LGB participants in this study feared that they would also have to conceal their sexual difference/identity and thus be forced into isolation in such circumstances to protect themselves from discrimination and hostility from staff and residents. Entering into care as a LGB&T individual is among the most significant anxieties about the future (Langley 2001; Stein and Almack 2012; Stonewall and Taylor 2012). Also, this current generation of old LGB&T people/care home residents may be reluctant to disclose their difference because they recall the hostility of much less ‘tolerant’ times marred by criminalisation, medicalisation and pathologisation (Dickinson 2015; Stein and Almack 2012).

With the above problems in mind, a quantitative study in Australia (Tolley and Ranzijn 2006) has recommended that exposure to non-heterosexual people, accurate knowledge about the diversity of their lived experiences and challenging heterosexism through training are needed to address heteronormativity among staff working in residential care facilities for older people. Also, in an Australian context and with older LGB&T service users in mind, Barrett and Stephens (2011) have challenged such thinking by emphasising a collective, systemic, organisational approach (rather than one based only on individualising responsibility) taken seriously by managers. This report, which constitutes an audit tool to assess LGB&T inclusivity, advocates consulting LGB&T staff and service users on specific needs and provision, educating providers through first-hand accounts of prejudice and discrimination, action-planning for change and ensuring the appropriate resources (including information) to implement any changes.

We would also concur with Hafford-Letchfield's (2008) view that the situation just described is as much connected with the structure and functioning of care homes as social systems as it is with individual care workers' responses to dominant discourse. Indeed, the point is not to blame staff or fellow residents, though we all need to take responsibility for our actions and expressed thoughts. Whilst there may be a general goodwill in an age of greater tolerance, there appears a sector-wide lack of strategic monitoring of the numbers of LGB&T residents and of institutional-wide initiatives and training designed to raise heterosexual staff *and* residents' awareness of the realities of sexual and gender difference (Ward *et al.* 2010).

As intimated, there has been some useful work on old(er) LGB&T experiences of care homes, though this is locally/regionally focused (Ward *et al.* 2010). For example, a report by Suffolk County Council and Suffolk LGBT Network (2012) highlights the marginalisation and invisibilisation of LGB&T residents. An in-depth interview-based study with older LGB&T individuals aged 50 plus in Northern Ireland, covering people in a range of care settings, has illuminated how many old(er) LGB&T people respond to discrimination by concealing their sexuality/sexual difference as far as possible. Again, this is strongly linked to fear of exclusion within the care home by residents as well as staff who, on account of ingrained heteronormativity, routinely fail to recognise the needs of individuals to stay in touch with LGB&T cultures, significant others and the sexual needs of LGB&T service users/residents (Rainbow Project and Age Northern Ireland 2011). As intimated, older LGB&T people are ‘twice hidden’, given the combined effects of ageism, homophobia/the heterosexual assumption. Further, as Doll (2012) has observed, going back into the closet can have considerable health implications and prevent professional carers from addressing the challenges faced by LGB&T residents.

The impetus for the above initiatives has come mainly from LGB&T individuals, academics, and/or health and social work professional organisations and staff employed in voluntary/campaigning organisations. Indeed, there are some nationally focused resources available. For example, Pugh *et al.* (2010) have provided a thoughtful training pack with case studies on inclusion of older LGB&T individuals as health and care service users. Another notable resource is a practical guide with examples of good practice for care homes and staff on involving individuals in meeting their own needs which comes complete with a checklist of various personal needs (Stonewall and Taylor 2012), which is also aimed at care home residents. The Commission for Social Care Inspection (2008) has also produced guidance for inspectors in relation to sexual orientation. This sets out best practice for LGB&T users of care services and how to support providers to maximise opportunities for all service users to ‘live up to their potential’. Whilst this and previous legal guidance covers legal duties, guidance for inspections on avoiding discrimination and securing equality of opportunity and outcomes, like more generic guidance, it fails to address sex and intimacy.

There also remain problematic knowledge/data gaps within academic research as well as care contexts (*see* Ward *et al.* 2005), which includes failure to recognise sexual difference, lack of suitable procedures and even physically removing the ‘problem’ represented by LGB&T residents from a communal space to placate the anxieties of heterosexual residents (Rosenfeld 2010). Furthermore, Phillips and Marks (2006) have drawn attention to how discourses operating in care settings exclude old(er) lesbian-identified women who face ‘triple invisibility’ on the (intersecting) grounds of age, gender and sexual difference in ‘asexual’ though still heterosexually defined spaces of care. This was compounded by habitual silencing and virtual erasure of lesbian identity and other non-normative sexualities and genders as evident in a lack of representation in promotional materials about services. It is not surprising then that those marked as sexually different describe their experiences of care homes as alienating (Phillips and Marks 2008). For these authors, breaking the silence requires further research, training to encourage professional carers to address *ingrained* ageism and homophobia, and a professional commitment to tackling injustice/inequality through anti-oppressive policies and practice. But, whilst critically focused on the distinct needs of older LGB&T users of health and care services, just like the generic literature on care homes, this body of work largely ignores residents' and older people's needs for sexual activity and/or intimacy.

## Concluding thoughts: practical recommendations and a critical future?

Concerns with physical independence and psychological functioning of older people and care home residents, whilst important, have tended to eclipse concern with sexuality/intimacy and old/er care residents. Whilst some literature on sexuality and older people and care homes has begun to emerge over the last 20 years or so, this is often more sexologically than sociologically focused and based more on quantitative than qualitative research methods. However, more recently some sociologically engaged work has highlighted how ageism or gerontophobia, at times combined with homo-/bi-/transphobia, can frustrate and/or marginalise the diverse sexual/intimate citizenship of residents. Some of this work has also usefully drawn attention to how care homes/settings can impose constraints on sexual self-expression and have intimated how good practice, currently rather piecemeal, might help overcome related problems.

In particular, we have drawn attention to the value of critical sociological, feminist–humanist-inspired, policy-engaged work that acknowledges old(er) care home residents'/individuals' exclusion from sexual/intimate citizenship and the discursive and/or structural impediments to meeting such needs in care homes. This work consciously avoids reinforcing notions of living in a care home as a necessarily reduced form of existence (Hafford-Letchfield 2008), thus opening up the possibility that homes could operate as more inclusive communities. However, recognising the sexual citizenship of LGB&T residents appears even more problematic and their needs concerning *sexual identification* have received much less scholarly and policy-based concern, though this has been partially rectified by practitioner-based and campaigning/voluntary organisations. Some research in this regard draws attention to how sexual and gender differences are habitually disregarded in care homes where older individuals feel pressured to conceal/deny sexual identities. However, whilst illuminating how disadvantage plays out for LGB&T individuals, even this body of work has little to say about older LGB&T service users' needs concerning sex and intimacy. The lack of a truly holistic approach to addressing sex, sexuality and intimacy in relation to older care home residents risks reinforcing older people's exclusion from sexual and intimate citizenship *per se*, but this issue takes on greater urgency in the case of LGB&T individuals.

There now appears a more critical body work like that just mentioned, which recognises old(er) people's and care home residents' erasure from sexual/intimate citizenship but consciously avoids framing this as a problem or pathology of individuals/residents requiring containment. This theme is particularly evident in the work of Hafford-Letchfield (2008), who draws attention to the need to tackle a range of biases, including ageism, sexism, ethnocentrism, ableism, heteronormativity and cisgenderism, that suffuse the literature on ageing sexuality and affect care practice. Hafford-Letchfield (2008) has proposed a set of practical solutions to the above issues which pivot around the valuing of differences. These include: avoiding heteronormative and cisgenderist assumptions about sexuality and sexual and gender identification in assessments; emphasising confidentiality, use of a range of images and positive responses towards human differences in service information/publicity; putting individuals in touch with culturally appropriate sexual health networks; and, crucially, consulting with users on their needs and wishes in an informed, sensitive way. In the specific context of care homes, it is recommended that care staff and systems should enable privacy for intimate experience, maintain choices around clothing/self-presentation, and that staff training and discussions concerning policy and practices should aim to balance consent and choice with safeguarding.

At the level of the care home, and in a rare interview-based study that involves care staff *and* residents across several care homes, Villar *et al.* (2014) have noted how residents may have discounted themselves as sexual/intimate citizens given loss of capacity, comparative lack of male partners, generational attitudes that associate sex with shame or equate it with youth, staff embarrassment about addressing sexual matters, and policing of sexual identity by fellow residents, relatives and staff. This study also offers important practical recommendations that concern opportunities for intimacy when residents' need for privacy is trumped by a quick access imperative, involving ‘open door’ or ‘no locked door’ policies. The authors also propose that sensitively delivered sex education could be made available and linked into discussion of health problems with residents; such issues requiring inclusion in guidance agreed between residents and professionals on meeting sexual/intimate and other needs. It has also been recommended that residents, should they choose, be able to access erotic aids, including pornography, and be encouraged to participate in group discussions, and that family members could also be supported to appreciate the sexual needs of older relatives (Hodson and Skeen 1994).

Any literature review is of necessity selective. This article is based on Anglophone literature that reflects North American, British and, even more substantially, Australian scholarship where ageing sexual citizenship has received greater attention and has been theorised in progressive ways better equipped to secure inclusion. We contend that some of our findings might apply in more economically developed countries. This suggests a fertile avenue for further research and especially in relation to lesser economically developed parts of the globe where poverty might trump consideration of sexuality and intimacy or, given the legacy of Empire, be difficult to talk about. This might apply to African countries (Anderson 2007) and Russia (Stella 2013), where those thought to represent putatively ‘abnormal’ sexual and gender difference risk punitive social and legal sanctions as well as their lives. Also, in many such countries, care homes, training and dedicated resources are unimaginable and cultural expectations dominate about looking after older parents/community members. However, societies like the Kalai society, Papua New Guinea, call into question the notion of sexual progressiveness as ‘Western’ when, rather than dismissing old(er) women as ‘past it’, as is common in consumerist societies, validate them as sexual beings (Hockey and James 1993). Closer to home, one further omission from this review and scholarship generally concerns engagement with the impact of material and organisational factors such as size of care homes, rates of retention/staff turnover, management and leadership style, and the client base; all of which affect the context of care and the decisions made on a day-to-day basis. Crucially, such factors are likely to affect care staff's and homes' abilities to meet the needs of residents, including those relating to sexuality.

Whilst the above issues need addressing, the limitations of this article are outweighed by the fact that we have addressed the need for thinking beyond bio-medical, book-keeping approaches to ageing sexuality. We have also identified a need to investigate and address practically the obstacles to dissemination and implementation of existing guidance across the spectrum of genders and sexualities. Whilst we support many of the recommendations offered in the more critical approaches to the topic at hand, we are also mindful of the need for research to go beyond problem-spotting to unearth actual good practice – the good news stories that could be disseminated across the care sector for older people.

Given increasing sexualisation of advanced capitalist cultures (Attwood 2009) – posing threats, contradictions and opportunities in certain parts of the globe – the issue of sexuality and intimacy for old people represents an increasingly important rights and health issue, nationally and internationally. In the Anglophone world, the sexuality of older people has been shaped by encounters with various liberation movements, which help us think beyond stereotypes of Vera Lynn and the War. In this broader historical context, there is a small but growing body of critical research that reminds us of the ongoing sexual/intimate needs of care home residents who, even if affected by dementia, need to play a meaningful part in decision-making (Bauer 1999; Deacon, Minchinello and Plummer 2006; Gott 2005; Hafford-Letchfield 2008; Villar *et al.* 2014). There are also encouraging signs that older people are appropriating the term ‘intimacy’, which augurs well. In some cases, this might indicate a pragmatic, compensatory response to loss of capacity but in other cases could signify a reversal of meaning of ageing sexuality on terms more convivial to older individuals. As researchers, practitioners or interested citizens, we need to play our part in advancing such an agenda.
